# An aberrant DNA methylation signature for predicting the prognosis of head and neck squamous cell carcinoma

**DOI:** 10.1002/cam4.4142

**Published:** 2021-07-27

**Authors:** Dayang Chen, Mengmeng Wang, Ying Guo, Wei Wu, Xiang Ji, Xiaowen Dou, Huamei Tang, Zengyan Zong, Xiuming Zhang, Dan Xiong

**Affiliations:** ^1^ Medical Laboratory Shenzhen Luohu People's Hospital Shenzhen China; ^2^ School of Medicine Anhui University of Science and Technology Huainan China; ^3^ Department of Clinical Laboratory Shanghai Public Health Clinical Center Fudan University Shanghai China

**Keywords:** DNA methylation, HNSCC, nomogram, prognosis, risk score

## Abstract

Head and neck squamous cell carcinoma (HNSCC) is a common malignancy worldwide with a poor prognosis. DNA methylation is an epigenetic modification that plays a critical role in the etiology and pathogenesis of HNSCC. The current study aimed to develop a predictive methylation signature based on bioinformatics analysis to improve the prognosis and optimize therapeutic outcome in HNSCC. Clinical information and methylation sequencing data of patients with HNSCC were downloaded from The Cancer Genome Atlas database. The R package was used to identify differentially methylated genes (DMGs) between HNSCC and adjacent normal tissues. We identified 22 DMGs associated with 246 differentially methylated sites. Patients with HNSCC were classified into training and test groups. Cox regression analysis was used to build a risk score formula based on the five methylation sites (cg26428455, cg13754259, cg17421709, cg19229344, and cg11668749) in the training group. The Kaplan–Meier survival curves showed that the overall survival (OS) rates were significantly different between the high‐ and low‐risk groups sorted by the signature in the training group (median: 1.38 vs. 1.57 years, log‐rank test, *p* < 0.001). The predictive power was then validated in the test group (median: 1.34 vs. 1.75 years, log‐rank test, *p* < 0.001). The area under the receiver operating characteristic curve (area under the curve) based on the signature for predicting the 5‐year survival rates, was 0.7 in the training and 0.73 in test groups, respectively. The results of multivariate Cox regression analysis showed that the riskscore (RS) signature based on the five methylation sites was an independent prognostic tool for OS prediction in patients. In addition, a predictive nomogram model that incorporated the RS signature and patient clinical information was developed. The innovative methylation signature‐based model developed in our study represents a robust prognostic tool for guiding clinical therapy and predicting the OS in patients with HNSCC.

## Graphical abstract

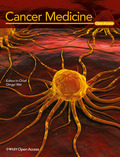

## 1 BACKGROUND

Head and neck squamous cell carcinoma (HNSCC) is the sixth most cancer worldwide and a collective term for cancers closely associated with squamous differentiation in the head and neck.^1^ HNSCC commonly mainly consists of a group of tumors derived from the mucosal surfaces of four major anatomical sites: oral cavity, pharynx, larynx, and sinonasal cavity.^2^ Owing to the lack of effective tools for clinical risk assessment and early diagnosis, HNSCC is often not discovered until it has progressed into advanced stages in more than half of the patients. According to a 2019 report, 600,000 new HNSCC cases are discovered per annum, with a mortality rate of over 50% worldwide.^3^ The latest statistics show that the incidence of HNSCC in China is 3.628%, suggesting an increasing trend and presentation at a younger age. The risk factors for pathogenesis of HNSCC include unique genetic backgrounds, tobacco and alcohol consumption, and viral infections, such as human papilloma virus (HPV) and Epstein–Barr virus.^4^, ^5^, ^6^, ^7^, ^8^ Despite the advances in chemo‐ and radiotherapeutic treatment modalities, the overall 5‐year survival rate for HNSCC remains low.^9^ Although treatments administered at an early stage are effective in terms of development of fewer lymphatic metastases, HNSCC is often diagnosed at advanced stages. Therefore, there is an urgent need to identify more effective diagnostic biomarkers to guide clinical therapy and prognostic evaluation to increase the survival rates of patient.

DNA methylation is an epigenetic modification that occurs at cytosine‐phosphate‐guanine (CpG) dinucleotides and is critical for cancer development and progression. This modification has been shown to play an essential role in regulating gene expression, RNA processing, and protein function. Some studies have demonstrated that aberrant DNA methylation is a common and early event in HNSCC that precedes malignant cell proliferation.^10^ As the DNA methylation genes are closely associated with tumor metastasis and invasion, detection of these genes with the high accuracy has immense significance in the early diagnosis of HNSCC.^11^, ^12^ With the development of high‐throughput technologies, a series of aberrant DNA methylation genes, including *p16*, *p15*, *p14*, *DAPK*, and *E*‐*cadherin*, have been identified as differentially expressed genes in HNSCC.^13^, ^14^, ^15^, ^16^ Recently, instead of using single methylation gene or combinations of multiple genes to evaluate the early diagnosis of HNSCC, researchers have begun to comprehensively investigate the methylation and expression profiles of genes involved in HNSCC and evaluate their predictive values in the prognosis of HNSCC. Ma et al. developed a four‐gene methylation signature consisting of *ZNF10*, *TMPRSS12*, *ERGIC2*, and *RNF215* to predict the survival outcomes of patients with HNSCC.^17^ The identification of tumor‐specific methylation sites is critical for the early detection and prognosis of cancer.^18^ These sites are usually overexpressed in cancer cells, unlike in normal cells. These findings indicate that such tumor‐specific methylation sites have considerable potential for cancer screening.^19^

To date, very little research has been conducted in the use of methylation sites as a signature for predicting the prognosis of HNSCC. To investigate potential DNA methylation sites associated with HNSCC survival, a novel model based on five methylation sites signature was established in the current study. This model was used to evaluate both the training and test groups of 512 patients with HNSCC, and improve survival prediction of HNSCC.

## 2 METHODS

### 2.1 Data retrieval and analysis

In this study, we utilized the tool of The Cancer Genome Atlas (TCGA)‐Assembler to download the clinical and DNA methylation data of patients with HNSCC collected from the Illumina Infinium Human Methylation 450 Platform (San Diego, CA) from TCGA databank (https://portal.gdc.cancer.gov/).^20^ Methylation information pertaining to 529 HNSCC tissue samples and 50 adjacent normal tissue samples was collected from the level three methylation database. These data were preprocessed by TCGA pipelines in the form of *β* values calculated as *M*/(*M* + *U*), where *M* represents the methylated probe intensity and *U* represents the unmethylated probe intensity. Approximately 512 samples from patients with intact medical records (gender, age, tumor grade, clinical stage, and vital status) and methylated information were randomized into two groups: a training group with 341 samples (to identify and construct prognostic biomarkers) and a test group with the remaining 171 samples (to verify the accuracy of the prognostic biomarkers).

### 2.2 Identification and functional enrichment analysis of the differentially methylated genes

The differentially methylated genes (DMGs) were screened using four steps as follows: First, we downloaded the TCGA‐HNSC.methylation450.tsv file, which contains genome‐wide methylation site *β* values. Second, we downloaded the probe_full_annotation.txt file from the website (http://lifeome.net/software/fastdma/annotation/probe_full_annotation.txt) that contains the location of the methylation site in relation to the gene. Third, we calculated the average methylation of β within a gene. Fourth, the R (Version:3.5.1) package limma was applied to compare the mean β value of genes between the 529 HNSCC tissue samples and 50 adjacent normal tissue samples. Genes with *p* values and false discovery rates < 0.05 were considered to be DMGs. Then the corresponding methylation sites of the DMGs were retrieved for further analysis. To further study the functions of survival‐related DMGs, we performed gene ontology (GO) and Kyoto Encyclopedia of Genes and Genomes (KEGG) pathway analysis (https://www.genome.jp/kegg/) to investigate the roles of all 22 DMGs based on the R package “clusterProfiler”. *p* < 0.1 was set as the cutoff criterion.

### 2.3 Construction of a five‐methylation site signature in the training group

Univariate Cox proportional hazards regression analysis was performed to identify the methylation sites related to the overall survival (OS) rate of patients’ by setting *p* < 0.05. The candidate markers were evaluated by Cox multivariate analysis to screen out the most effective predictive diagnostic and prognostic sites, which were subsequently used to construct the following model to assess the prognosis risk:

$$\text{Riskscore (RS)}=\sum_{i}^{N}\left(\text{expression}*\text{coefficient}\right)$$

The Riskscore (RS) represents the risk score of patients with HNSCC, and *N* denotes the representative number of prognostic methylation sites. Expression refers to the expression value of methylation sites, and the coefficient refers to the regression coefficient of methylation sites, representing the contribution of methylation sites to the prognostic RS. Patients were separated into high‐ and low‐risk groups using the median RS from the training group as the cutoff point. Subsequently, a RS system was established, in which patients with an RS value higher than the median were assigned to the high‐risk group, whereas those with RS values lower than the median were assigned to the low‐risk group.

### 2.4 Statistical analysis

Kaplan–Meier survival curves were calculated using the R software's survival package to estimate the survival time and compare the survival probabilities between the high‐risk and low‐risk groups.^21^, ^22^ Subsequently, the time‐dependent receiver operating characteristic (ROC) curve was applied to assess the specificity and sensitivity with the survival prediction RS of the five methylation sites in the training group. The area under the curve (AUC) was then calculated. Prognostic DNA methylation site signatures were constructed by comparing the AUC values in the training group. Subsequently, the prognostic performance of the five‐methylation site signature was assessed in the test group based on the AUC values and results of the Kaplan–Meier survival analysis. The evaluated association between the expression levels of the five methylation sites and possibility of patient survival was constructed using a nomogram in the training group based on multivariable analysis.

### 2.5 Development of the predictive nomogram model

Of the 512 samples from patients with HNSCC, 289 were randomly selected based on the medical records to develop a predictive nomogram as per the “Iasonos” guidelines,^23^, ^24^ to predict the 1‐, 2‐, and 3‐year survival rates of patients with HNSCC. Nomogram was established by combining RS with clinical variables (age, gender, grade, and state) using a multivariable Cox regression model. In this model, Cox regression was performed to identify whether RS was an independent survival predictor to assess the survival rate of patients with HNSCC. The above‐mentioned analyses were conducted using the R program (version 3.5.1): survival random forest, limma, and ROC packages (Bioconductor, http://www.bioconductor.org/). Statistical analysis was also performed using the R program, and statistical significance was set at *p* < 0.05.

## 3 RESULTS

### 3.1 Patient characteristics

**FIGURE 1:**
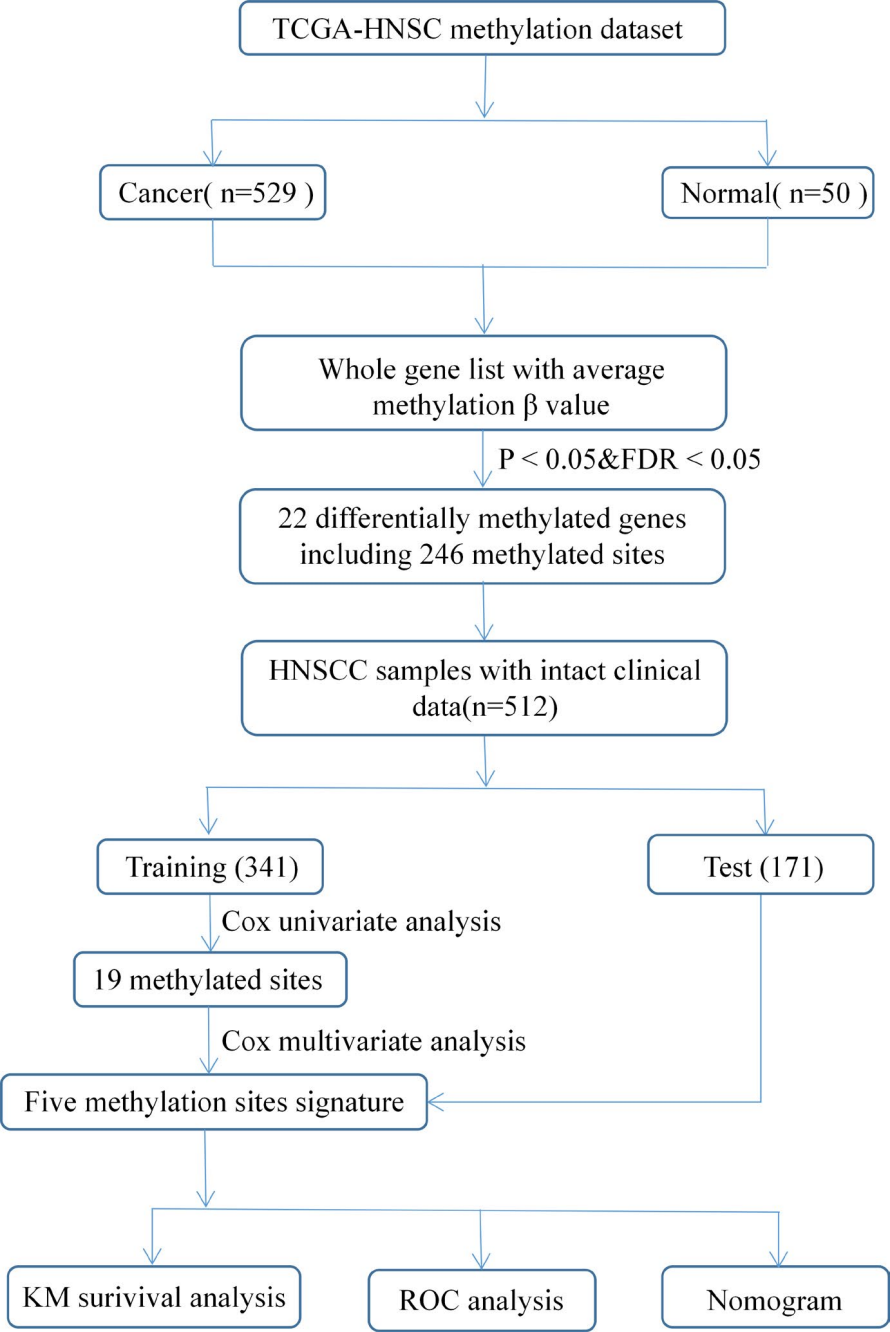
The flow chart of the study

A total of 528 patients with HNSCC were enrolled in this study. Based on the tumor‐node‐metastasis (TNM) staging system classification, the clinical stage of the tumors were classified into stages I–IV. Gender, age, grade, stage, and vital status were introduced as variables in this study. Patients lacking records for any one of these variables were excluded from the study. A total of 512 patients with HNSCC were randomly divided into training and test groups comprising of 341 and 171 patients, respectively. The clinicopathological characteristics of the 512 patients with HNSCC categorized by stage are summarized in Table 1. The tumor origin information of the 579 tissues from the TCGA database are shown in Table 2. Schematic of the technical route used in this study is illustrated in Figure 1. The five methylation sites that are closely associated with the OS of patients with HNSCC were identified in the training group and validated in the test group.

**TABLE 1:** Summary of patient demographics and characteristics

Characteristic	Training (*N* = 341)	Test (*N* = 171)
Gender		
Female	93 (27.3%)	43 (25.1%)
Male	248 (72.7%)	128 (74.9%)
Age (years)		
<60	146 (42.8%)	84 (49.1%)
≥60	195 (57.2%)	87 (50.9.%)
Grade		
1	41 (12.0%)	17 (10.0%)
2	204 (59.8%)	100 (58.5%)
3	76 (22.3%)	43 (25.1%)
4	3 (0.8%)	4 (2.3%)
Stage		
I	15 (4.4%)	12 (7.0%)
II	42 (12.3%)	26 (15.2%)
III	114 (33.4%)	25 (14.6%)
IV	181 (53.1%)	80 (46.8%)
Vital status		
Living	214 (62.6%)	106 (61.9%)
Dead	127 (37.4%)	65 (38.1%)

**TABLE 2:** The origins information of HNSCC and adjacent normal tissues from TCGA database used in this research

Site	HNSCC	Normal
Border of tongue	1	0
Mandible	1	0
Palate	1	0
Pharynx	1	0
Posterior wall of oropharynx	1	0
Retromolar area	1	0
Supraglottis	1	0
Upper gum	1	0
Ventral surface of tongue	1	0
Anterior floor of mouth	2	0
Lower gum	2	0
Lip	3	0
Hard palate	4	1
Gum	8	0
Hypopharynx	9	0
Oropharynx	9	0
Cheek mucosa	19	0
Mouth	22	0
Base of tongue	24	2
Tonsil	47	0
Floor of mouth	55	4
Overlapping lesion of lip, oral cavity, and pharynx	70	5
Larynx	116	16
Tongue	130	22

Abbreviations: HNSCC, head and neck squamous cell carcinoma; TCGA, The Cancer Genome Atlas.

### 3.2 Functional enrichment analysis of DMGs in HNSCC

**FIGURE 2:**
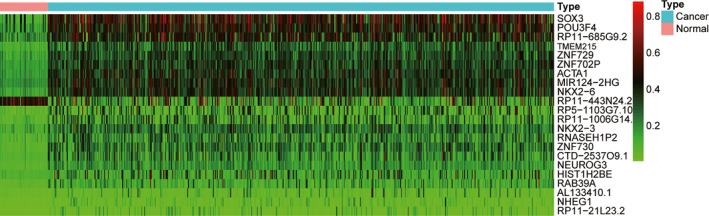
Heat map of the differentially expressed methylated genes between HNSCC samples and corresponding normal tissues. HNSCC, head and neck squamous cell carcinoma

**FIGURE 3:**
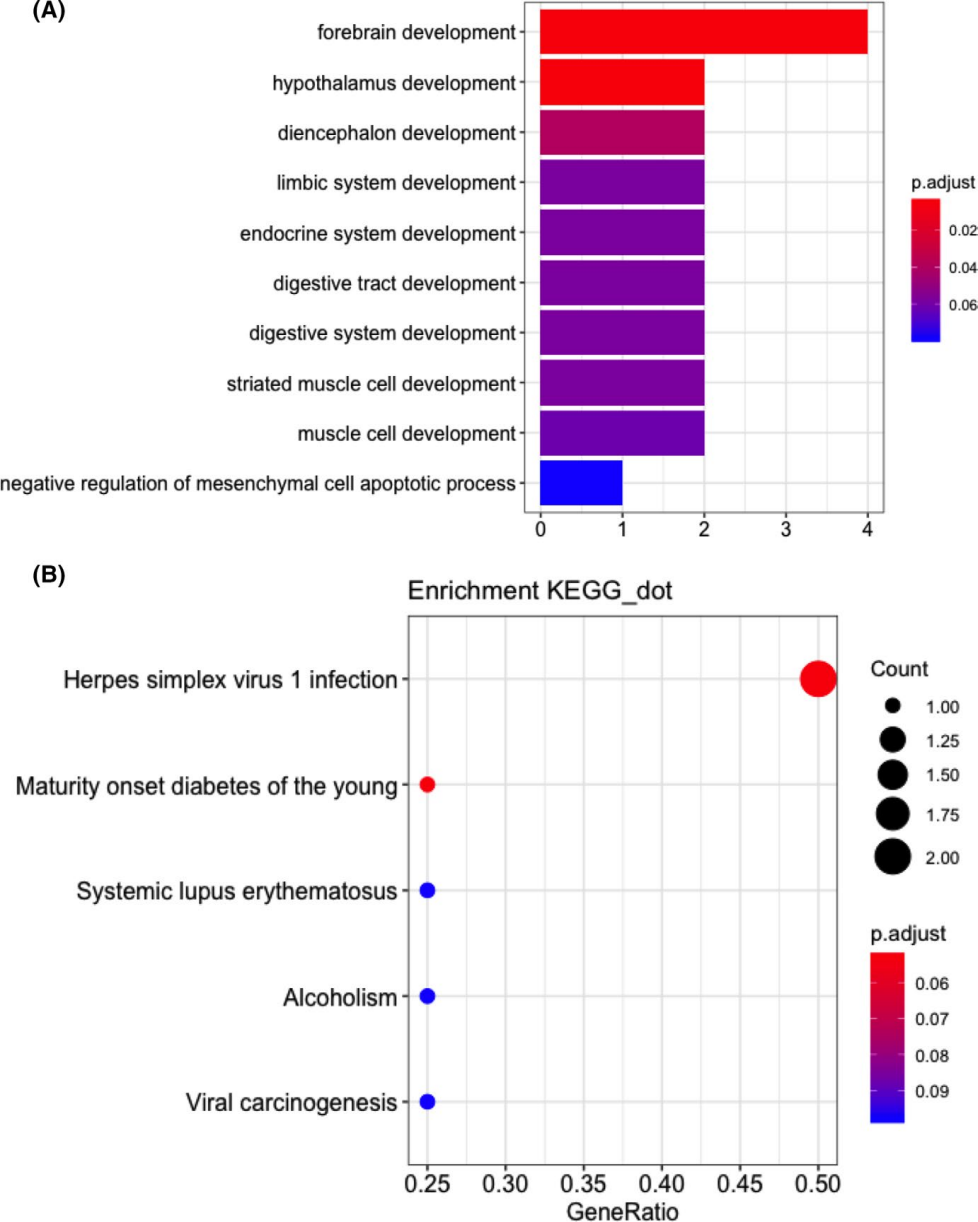
GO (A) and KEGG (B) pathway enrichment analysis of 22 DMGs involved in HNSCC. The top significant terms were shown in the heat map. Different color indicates the *p* value of different items. DMG, differentially methylated gene; GO, Gene Ontology; HNSCC, head and neck squamous cell carcinoma; KEGG, Kyoto Encyclopedia of Genes and Genomes

We identified 22 DMGs in the methylation profile (Table 3), corresponding to 246 methylated sites (Figure 2). To gain further insights into the potential functions of the 22 identified DMGs, GO and KEGG pathway analyses were performed to investigate their biological functions. The top significant terms of in the GO analysis (Figure 3A) showed that the DMGs involved in HNSCC were mainly enriched in “forebrain development,” “hypothalamus development,” and “diencephalon development” terms, which indicated that the DMGs may play a vital role in head and neck development. KEGG analysis (Figure 3B) showed that the DMGs were enriched in pathways associated with carcinogenesis, such as “Herpes simplex virus 1 infection” and “viral carcinogenesis.”

**TABLE 3:** The twenty‐two differentially methylated genes

Gene	Normal mean *β* value	Tumor mean *β* value	logFC	*p* value
TMEM215	0.1127	0.2978	1.4020	7.94E − 26
CTD‐2537O9.1	0.0860	0.2075	1.2716	3.44E − 14
NKX2‐3	0.1255	0.2572	1.0354	1.11E − 17
AL133410.1	0.0284	0.0608	1.0995	2.45E − 05
RP11‐443N24.2	0.4964	0.2472	−1.0057	4.11E − 15
HIST1H2BE	0.0985	0.1977	1.0049	1.48E − 10
NHEG1	0.0187	0.0442	1.2397	1.84E − 06
SOX3	0.1827	0.4806	1.3955	2.63E − 22
NEUROG3	0.0722	0.1844	1.3517	1.97E − 26
ACTA1	0.1714	0.3741	1.1259	1.02E − 29
MIR124‐2HG	0.1937	0.3925	1.0191	8.97E − 29
RP11‐1006G14.2	0.0781	0.1791	1.1982	2.33E − 06
POU3F4	0.2028	0.4409	1.1205	7.42E − 24
RP11‐21L23.2	0.0124	0.0408	1.7158	0.002556851
ZNF730	0.0722	0.2428	1.7492	1.26E − 24
RAB39A	0.0576	0.1429	1.3111	8.71E − 20
NKX2‐6	0.1501	0.3851	1.3589	4.55E − 30
RNASEH1P2	0.0883	0.2091	1.2439	1.62E − 13
RP5‐1103G7.10	0.0619	0.1709	1.4648	3.13E − 06
RP11‐685G9.2	0.2024	0.4104	1.0198	1.20E − 19
ZNF729	0.1480	0.3288	1.1517	8.28E − 26
ZNF702P	0.1668	0.3503	1.0703	3.04E − 28

### 3.3 Selection of candidate prognostic methylated sites in the training group

**FIGURE 4:**
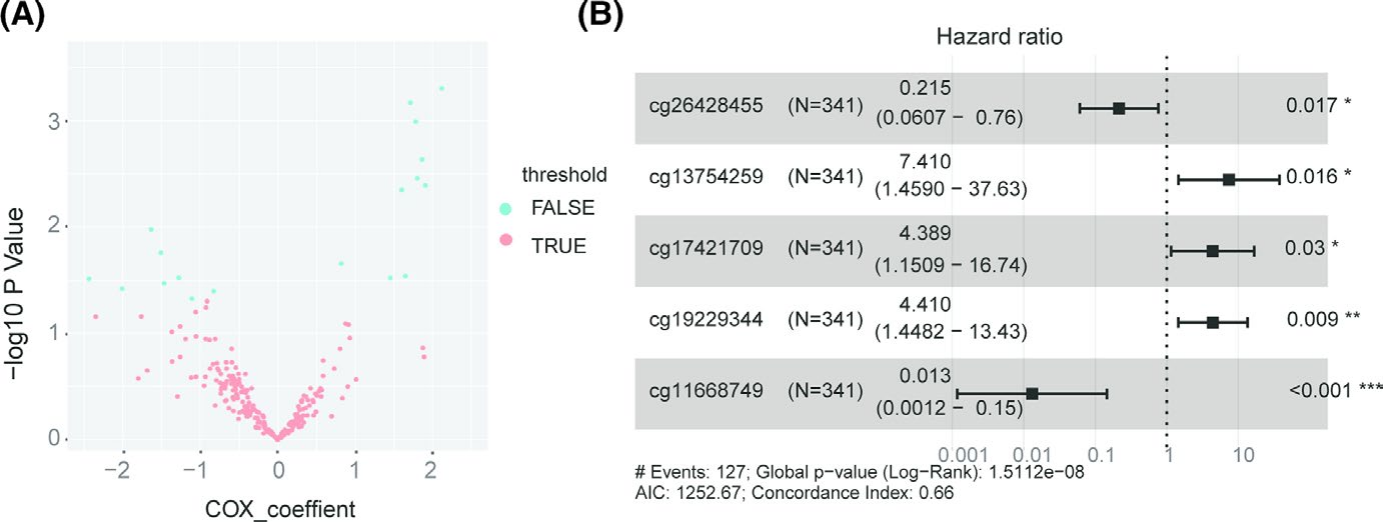
Univariate and multivariable Cox analyses. (A) Cox univariate analyses, 19 methylation sites were selected by *p* < 0.05. (B) Cox multivariate analyses

We then screened 512 patients with complete clinical information, including gender, age, and clinical stage, and randomly divided them into a training group (*n* = 341) and a test group (*n* = 171). Univariate Cox proportional hazards regression analysis was used to explore the relationship in the training group between OS and 246 differentially methylated sites identified above. A total of 19 methylated sites were found to be significantly correlated with OS (*p* < 0.05, Figure 4A). The most predictive differentially expressed methylated sites that were strongly correlated with patient survival were then screened out from the 19 methylation sites using Cox multivariate analysis. An optimal predictive model for HNSCC prognosis, composed of cg26428455, cg13754259, cg17421709, cg19229344, and cg11668749 was constructed with the minimum Akaike information criterion, *p* < 0.05, and Harrel's concordance index (*C*‐index) of the model for OS prediction of 0.66 (see Figure 4B). Three methylation sites were presumed to be risk factors (hazard ratio [HR] > 1), whereas two were protective factors (HR < 1) and the HR of cg11668749 was maximal (0.0012–0.15).

### 3.4 Building a predictive DNA methylation site signature

To investigate the performance of the five methylation sites in predicting recurrence, we calculated the RS of the five‐site signature of all the patients in the training group using the RS formula as follows:

RS=(‐1.54×methylation level of cg26428455)+(2.00×methylation level of cg13754259)+(1.48×methylation level of cg17421709)+(1.48×methylation level of cg19229344)+(‐4.33×methylation level of cg11668749)

**FIGURE 5:**
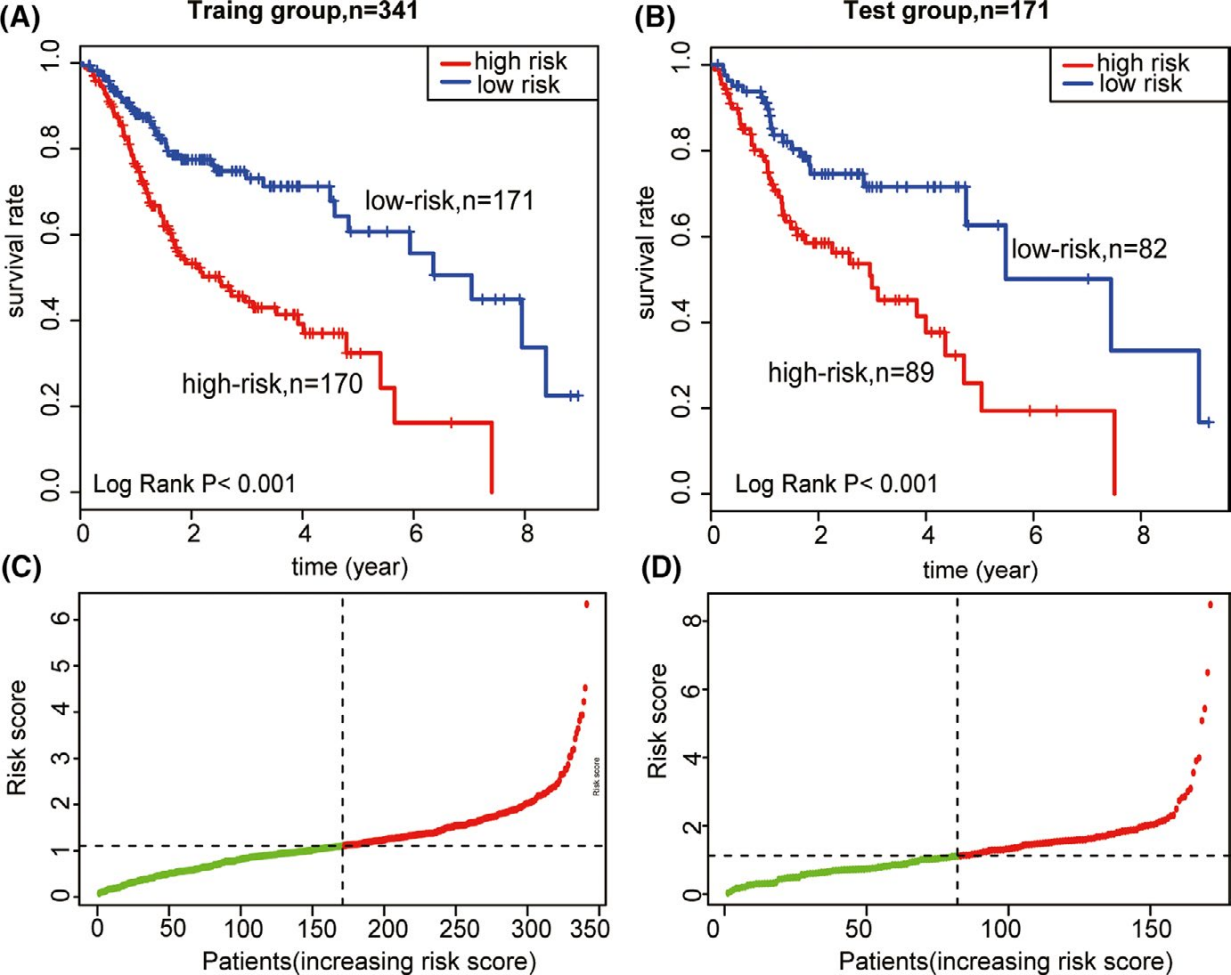
Kaplan–Meier survival analysis and RS distribution. (A) The Kaplan–Meier survival analysis of OS for TCGA training group (*n*= 341). (B) The Kaplan–Meier survival analysis of OS for TCGA test group (*n* = 171). (C) Five methylation sites RS distribution in training group (*n* = 341). (D) Five methylation sites RS distribution in test group (*n* = 171). OS, overall survival; RS, riskscore; TCGA, The Cancer Genome Atlas

Using the median RS as the cutoff point, the patients in the training group were divided into either a high‐risk group or low‐risk group. As the median of RS in the training group was 1.11, the patients with RS > 1.11, were assigned to the high‐risk group and the others with RS ≤ 1.11 were assigned to the low‐risk group. The survival status and distribution of the training group are shown in Figure 5A. The results of Kaplan–Meier survival analysis indicated that the patients in the low‐risk group were correlated with a higher survival rate than those in the high‐risk group (*p* < 0.001). The prognostic RS of 171 patients was simultaneously calculated in the test group to confirm the predicted results of the training group. Following the same logic, these patients were also divided into the high‐ and low‐risk groups based on the RS median from the training group. The survival rate of patients in the low‐risk group was also significantly higher than that of patients in the high‐risk group (Figure 5B), which was in accordance with the findings from the training group. The RS distribution was identical between patients in the training and test groups (Figure 5C,D).

### 3.5 Building a predictive nomogram based on the five‐methylation site signature

**FIGURE 6:**
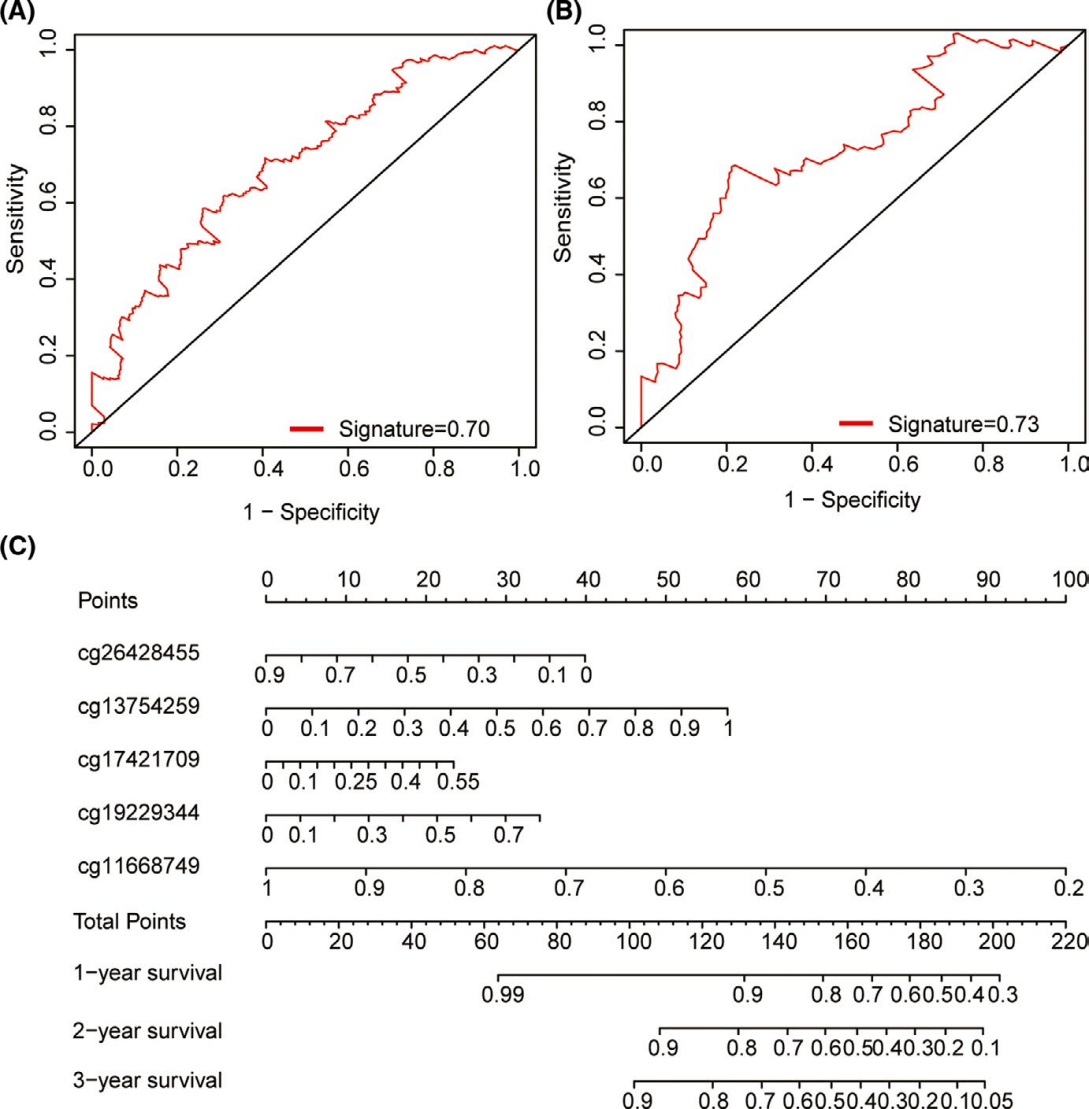
Build a predictive nomogram model of five methylation sites signature for 1‐, 2‐, and 3‐year OS of HNSCC. (A) ROC curve for the 5‐year survival prediction in training group. (B) ROC curve for the 5‐year survival prediction in test group. (C) Nomogram for predicting 1‐, 2‐, and 3‐year OS of HNSCC using five methylation sites. Each patient in training group was assigned to a score on the point scale. By summing up the total points of the five methylation sites lie on “Total Points” axis, we could predict the probability of 1‐year and 3‐year OS rate plotted on the three axes below. HNSCC, head and neck squamous cell carcinoma; OS, overall survival; ROC, receiver operating characteristic

To investigate the prognostic value of the five‐methylation site signature, a separate ROC analysis was performed in the training and test groups because a larger AUC of ROC offers a better model for prediction. The results showed that the AUC was 0.70 in training group, indicating that the signature has a good ability to predict the survival of patients with HNSCC (Figure 6A). The signature was then confirmed in the test group (AUC = 0.73), indicating that this signature potentially represents a robust prognostic biomarker for HNSCC (Figure 6B). We established a nomogram for OS prediction in HNSCC based on the five‐methylation site signature. Independent prognostic predictors, including cg26428455, cg13754259, cg17421709, cg19229344, and cg11668749, were integrated into a nomogram, where the probability of 1‐, 2‐, and 3‐year survival of patients with HNSCC was calculated using the formula of Exp * Coef for each site (Figure 6C). “Exp” refers to the degree of DNA methylation, and “Coef” represents the corresponding Cox regression coefficient. The result of the concordance index based on the multivariate analysis of the five‐methylation site signature (0.66) clearly showed that the nomogram had adequate prognostic power to predict the OS of patients with HNSCC.

### 3.6 Building a nomogram consisting of clinical data and RS

**FIGURE 7:**
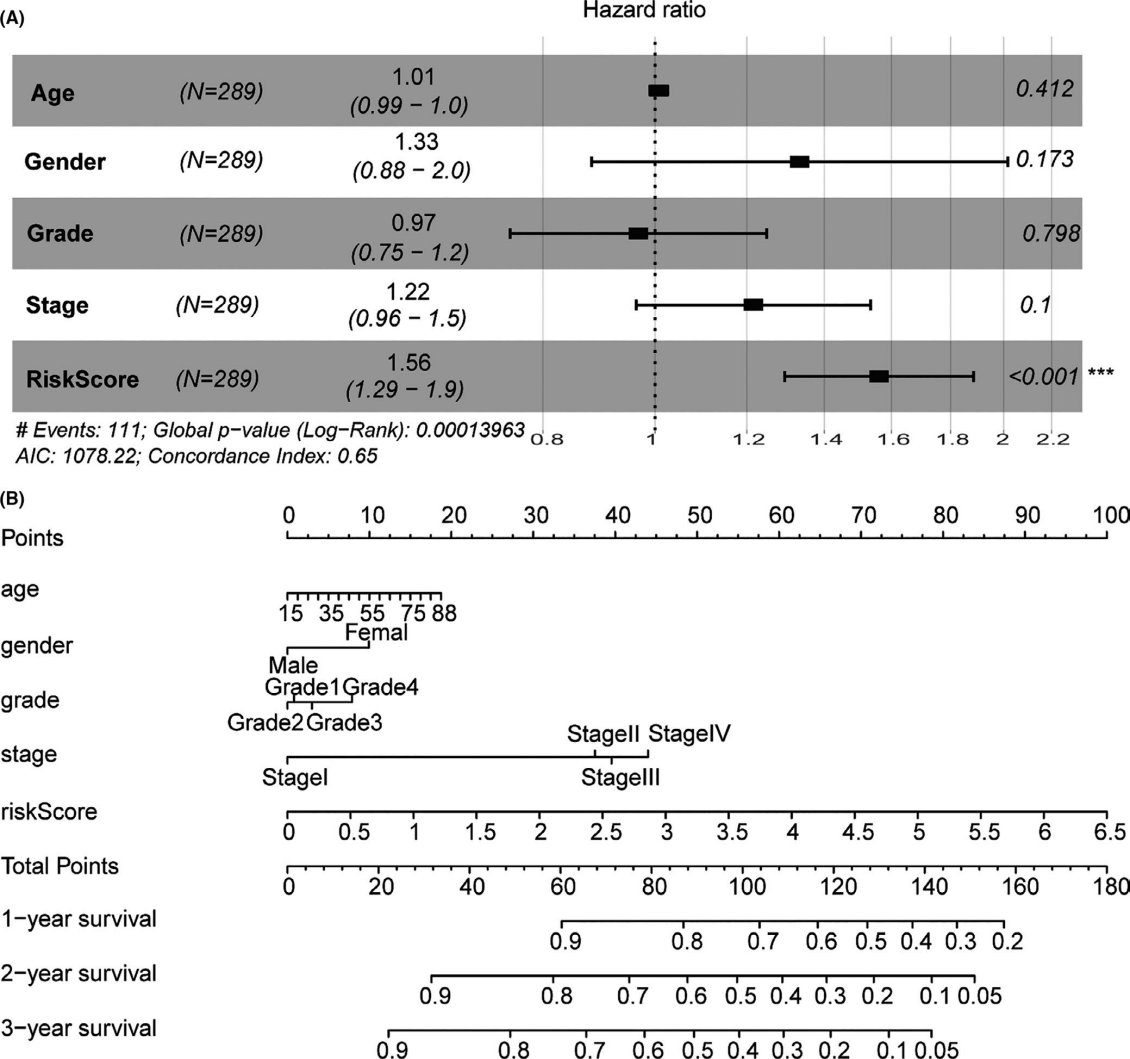
Establishment of a nomogram for OS prediction in HNSCC. (A) Multivariate analysis revealed a significant association between the RS and OS (HR = 1.56, *p* < 0.001). (B) A nomogram combining clinical data (age, gender, grade, and stage) with RS provides great guiding value for predicting 1‐, 2‐, and 3‐year OS of HNSCC. HNSCC, head and neck squamous cell carcinoma; HR, hazard ratio; OS, overall survival; RS, riskscore

A multivariate Cox regression analysis was performed in a cohort of 289 patients, in which age, gender, grade, and stage were set as the co‐variables, to determine whether the prognostic value of RS was an independent variable among the other clinical inputs. The multivariate analysis showed that the RS (HR = 1.56, 95% confidence interval = 1.29–1.9, *p* < 0.001) was independently associated with the OS of patients with HNSCC in the multivariate analyses (Figure 7A). A prognostic nomogram based on the multivariate analysis results was formulated to establish an effective method to predict the probabilities of 1‐, 2‐, and 3‐year OS in HNSCC, with a concordance index of 0.65. A predictive nomogram was also established in which the score integrated the other four independent prognostic variables including age, gender, grade, and stage (Figure 7B), and each variable received a point corresponding to the point axis upward. Then, the 1‐, 2‐, and 3‐year survival rates were estimated by the total points downward. Patients with complete clinical information would obtain total points reflecting the probability of 1‐, 2‐, and 3‐year survival. The prognostic nomogram showed that the RS provides a better guiding value for clinical prognosis of HNSCC followed by stage.

## 4 DISCUSSION

Head and neck squamous cell carcinoma is a broad category of carcinomas arising in the nasal cavity, oral cavity, pharynx, larynx, thyroid, and esophagus.^25^ As HNSCC is often diagnosed at a very advanced stage, more sensitive prognostic approaches are urgently needed. Currently, the traditional prognostic determinant is based on the TNM staging system; however, its clinical outcome differs greatly among patients, even at the same stage. Therefore, the TNM staging system is not sufficient for personalized treatment because of the unpredictable clinical outcomes among patients.^12^ Over the past few years, several powerful predictive biomarkers have been reported to improve the clinical management of HNSCC. DNA methylation is a well‐known epigenetic modification that alters the expression of key tumorigenesis‐associated genes without any alteration in the genetic sequence.^26^ These modifications have been shown to be closely related to the occurrence and development of cancers, and many related biomarkers have been reported.^27^, ^28^ Tumor‐specific methylated sites are critical for the accurate diagnosis of cancers.^29^ As aberrant DNA methylation has been shown to be associated with HNSCC tumorigenesis, it may serve as novel predictive biomarkers for the prognosis in HNSCC.^30^, ^31^ DNA methylation genes, including *p16*, *p15*, *p14*, *DAPK*, and *E*‐*cadherin*, associated with HNSCC tumorigenesis have been reported in previous studies.^32^, ^33^ Schröck et al. revealed that DNA methylation of *SHOX2* and *SEPT9* is associated with HNSCC development through a prospective cohort study.^30^ In this study, we found that DNA methylation may be used for HNSCC diagnosis, staging, risk stratification, and monitoring. Sailer et al. reported that DNA methylation of *PITX3* was an independent prognostic biomarker of OS prediction in patients with HNSCC, and the methylation gene was used to process the risk stratification for individualized treatment.^31^ These findings suggest that the evaluation of aberrant DNA methylation genes may potentially serve as biomarkers for the diagnosis or prognosis of HNSCC.

In this study, we collected and analyzed the methylated genes and expression profiles of patients with HNSCC from the TCGA database and identified 22 DMGs corresponding to 246 methylated sites identified above. Through a series of statistical analyses, a RS signature composed of five methylated sites (cg26428455, cg13754259, cg17421709, cg19229344, and cg11668749) related to the prognosis of HNSCC was verified. We then constructed a visualized nomogram model that combined age, gender, grade, and TNM stage as covariates with the RS signature to predict, the 1‐, 2‐, and 3‐year OS of patients with HNSCC. Ma et al. developed a four‐gene methylation signature consisting of *ZNF10*, *TMPRSS12*, *ERGIC2*, and *RNF215*. Their work demonstrated that this signature could predict the survival outcomes of patients with HNSCC and provided a potential therapeutic target for HNSCC therapy.^17^ Another study by Pan et al. screened six methylation‐driven genes related to the prognosis of HNSCC, and constructed a linear risk model with the six genes, they also revealed that all six genes may be used as independent prognostic markers and represented potential drug targets.^34^ Few studies have previously reported the prognostic signatures based on methylation sites in HNSCC. Therefore, we conducted the present study to investigate the prognostic value of methylated sites in HNSCC. The prognostic nomogram model of HNSCC established in our study comprehensively considered the clinical information of patients, and its visualization characteristics were conducive to guide clinical judgment regarding the prognosis of patients.

In this study, a RS signature was established using five methylation sites that were mapped to four DMGs, including *NEUROG3* (*cg26428455*), *ACTA1* (*cg11668749*), *NKX2*‐*3* (*cg19229344*, *cg13754259*), and *RP11*‐*1006G14*.*2* (*cg17421709*). Among these DMGs, *NEUROG3*, *ACTA1*, and *NKX2*‐*3* were already annotated in earlier studies, whereas *RP11*‐*1006G14*.*2* was unknown. *NEUROG3* was found to be essential for endocrine differentiation in the pancreatic endocrine lineage and shown to be the earliest marker specific to pancreatic endocrine lineage.^35^ Recently, a bioinformatic analysis based on the aberrantly methylated differentially expressed genes and pathways in colorectal cancer (CRC) showed that *ACTA1* may serve as an aberrant methylation‐based biomarker for precise diagnosis and treatment of CRC.^36^ *NKX2*‐*3*, another DMG related to HNSCC is one of the most critical epigenetic markers associated with the pathogenesis of human melanoma cell lines.^37^ *NKX2*‐*3* was found to be upregulated in B cell lines and intestinal tissues from patients with Crohn's disease and downregulated in CRC.^38^, ^39^, ^40^ Among the four DMGs, only *ACTA1* was previously reported to be an early detectable marker in certain tumors and a potential prognostic biomarker.^41^, ^42^, ^43^, ^44^, ^45^ Yang et al. revealed that *ACTA1* is crucial for regulating the occurrence and progression of HNSCC, and represents a potential target for individual clinical treatment.^42^ Our study provides a context for further investigations into the functions of the four DMGs.

The stage‐specific survival time of HPV + HNSCC is significantly longer than that of HPV − HNSCC.^46^ HPV has been identified as a risk factor for tumors in the oropharyngeal subsite.^47^ We reviewed the HPV status of TCGA HNSCC dataset. Our data showed that the HPV‐negative group had a higher RS and lower survival rate than the HPV‐positive group (Figure S1A,B). HNSCCs arising from different origins, therefore, we compared the predictive value of the prognostic model in HNSCCs of different origins between the two main subtypes of HNSCC (larynx and tongue cancer) in the TCGA database. As shown in Figure S1C,D, the survival rate between the two subtypes was not significantly different. However, we did not include tumor site and HPV status as clinical covariates for the following reasons. First, 89 cases with HPV status accounted for a low proportion of the total number of samples and consisted of different tumor sites (Table S1). Although the survival time was increased with HPV positivity in pharyngeal and oropharyngeal cancer, the available sample types and size do not provide tangible evidence for the other HNSCC subtypes. Second, HPV detection is not a routine test for all HNSCC subtypes. If HPV status were included, the nomogram would appear impractical. Tumor site does not show a univariate relationship with outcome and is not included in the multivariate Cox proportional hazards regression model. Third, the four routine clinical variables were carefully chosen, given the number of events available, to ensure parsimony of the final nomogram.

## 5 CONCLUSIONS

In this study, five methylation sites (cg26428455, cg13754259, cg17421709, cg19229344, and cg11668749) related to four DMGs (*NEUROG3*, *ACTA1*, *NKX2*‐*3*, *and RP11*‐*1006G14*.*2*) were identified and used to develop a prognostic model for HNSCC. Our study not only provides an approach for predicting the OS rate of patients with HNSCC, but also a new prospect for evaluating the clinical treatment outcomes of HNSCC. Although the calculated RS based on the five‐methylation site signature combined with the clinical data provides a valuable predictive model for the prognosis of HNSCC, there are still some limitations in this study. Obtaining large number of clinical samples, especially from the nasopharyngeal site remains a challenge. It should be noted that the potential mechanisms underlying the presence of prognostic methylation sites in HNSCC remain unknown and further studies are needed to investigate their functional roles in tumors. In addition, the accuracy and robustness of the prognostic signature needs to be confirmed in clinical practice.

## CONFLICT OF INTERESTS

The authors declare that they have no competing interests.

## AUTHOR CONTRIBUTIONS

Conceived and designed the experiments: Dayang Chen, Mengmeng Wang, Ying Guo, Xiuming Zhang and Dan Xiong. Literature research and data acquisition: Ying Guo, Wei Wu and Xiang Ji. Designed analysis framework: Dayang Chen, Xiaowen Dou and Dan Xiong. Data analysis and interpretation: Mengmeng Wang, Ying Guo, Huamei Tang and Xiuming Zhang. Manuscript submission and revision: Zengyan Zong, Dayang Chen and Mengmeng Wang. Manuscript writing: All authors. Final approval of manuscript: All authors.

## Supporting information

FIGURE S1

Table S1

## Data Availability

All the clinical and the DNA methylation data of HNSCC patients used in this research can be retrieved from the Cancer Genome Atlas (TCGA) data bank (https://portal.gdc.cancer.gov/). And the datasets generated or analyzed in this manuscript are available from the corresponding author upon reasonable request.
