# Supplementary figures and images for: An aberrant DNA methylation signature for predicting the prognosis of head and neck squamous cell carcinoma
Source: Cancer Med. 2021 Jul 27;10(17):5936–47. doi: 10.1002/cam4.4142 (PMC8419750; doi:10.1002/cam4.4142)

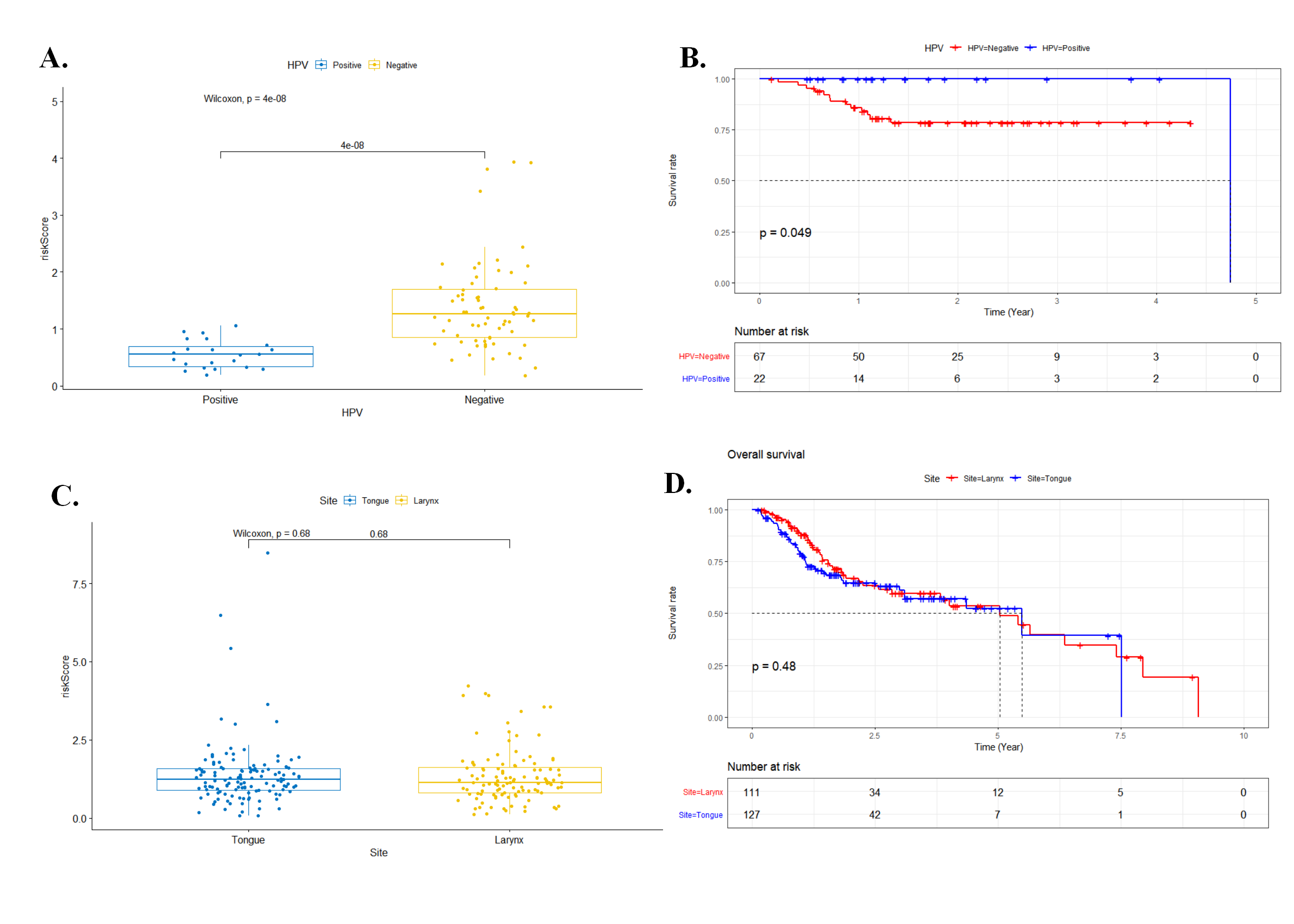

Supplement: Supplementary file 1 — FIGURE S1 [file CAM4-10-5936-s001.tif]
