# Supplementary material for: An aberrant DNA methylation signature for predicting the prognosis of head and neck squamous cell carcinoma
Source: Cancer Med. 2021 Jul 27;10(17):5936–47. doi: 10.1002/cam4.4142 (PMC8419750; doi:10.1002/cam4.4142)
Supplement: Supplementary file 2 — Table S1 [file CAM4-10-5936-s002.docx]

**Table S1. The origins information of HNSCC tissues with HPV result.**

| **Site** | **HPV Negative** | **HPV Positive** |
| --- | --- | --- |
| Base of tongue | 2 | 4 |
| Cheek mucosa | 2 | / |
| Floor of mouth | 9 | / |
| Gum | 3 | / |
| Hard palate | 1 | / |
| Hypopharynx | 2 | 1 |
| Larynx | 15 | / |
| Mouth | 2 | / |
| Oropharynx | 2 | / |
| Overlapping lesion of lip, oral cavity and pharynx | 6 | 1 |
| Posterior wall of oropharynx | 1 | / |
| Supraglottis | 1 | / |
| Tongue | 20 | / |
| Tonsil | 1 | 16 |
